# Supplementary material for: Binaphthyl-Based Chiral Macrocyclic Hosts for the Selective Recognition of Iodide Anions
Source: Molecules. 2023 Jun 15;28(12):4784. doi: 10.3390/molecules28124784 (PMC10303813; doi:10.3390/molecules28124784)
Supplement: Supplementary file 1 [file molecules-28-04784-s001.zip › molecules-2447614-supplementary.pdf]

Supporting Information

Binaphthyl-based chiral macrocyclic hosts for selective recognition of iodide anion

Zong-Cheng Wang[1], Ying-Zi Tan[1], Lin-Li Tang[1] and Fei Zeng[1,]*

[1]Department of Biology and Chemistry, Hunan University of Science and Engineering, Yongzhou 425199, China.

E-mail: zengfei@iccas.ac.cn

Contents

1. **Materials and Methods...2**
2. **Synthesis of New Compounds...2**
3. **^{1}H NMR and ^{13}C NMR Spectral of New compounds. 4**
4. **^{1}H NMR studies of Complexation of the Host and Guest 9**
5. **Determination of the Association Constants of the Complexes..............10**
6. **ESI MS studies of new compouds and complexes....................................16**

1. Materials and Methods.

All reactions were carried out with oven-dried glassware. Commercial reagents were used without further purification. Flash column chromatography was performed on 100-200 mesh silica gel. ^{1}H NMR, ^{13}C NMR spectra were recorded on a Bruker DMX400 NMR spectrometer. Melting points were determined using WRR melting point apparatus and were uncorrected. High Resolution atmospheric-pressure chemical ionization mass spectra (APCI-MS) were determined by Bruker Daltonics. Inc, APEX II. FT-ICRMS. Electrospray ionization mass spectra (ESI-MS) were recorded on the Thermo Fisher® Exactive LC-MS spectrometer.

2. Synthesis of New Compounds.

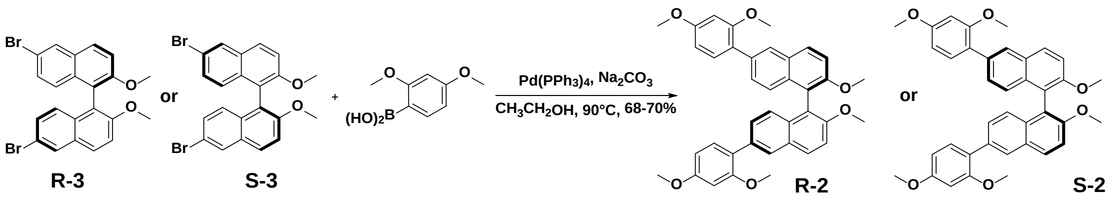

Compound R-2: A mixture of R-3[S1] (4.70 g, 10 mmol), Na_2CO_3 (2.96 g, 28 mmol), 2,4-dimethoxyphenylboronic acid (4.00 g, 22 mmol), and catalytic amount of CuI (21 mg) and tetrakis(triphenylphosphine)- palladium (320 mg) in 100 mL CH_3CH_2OH in a flask was stirred at 90 ^{o}C for 24 h under N_2. After evaporating the solvents, resulting mixture was extracted with dichloromethane (3 × 50 mL) and then washed with water and brine successively. The organic layer was dried over anhydrous Na_2SO_4 and evaporated. The residue was purified by column chromatography on silica gel with dichloromethane/ Petroleum ether as eluent (4:1) to afford compound **R-2** (3.99 g, yield 68%) as a yellow solid. ^{1}H NMR (400 MHz, Chloroform-*d*) δ 8.01 (d, J = 10.3 Hz, 4H), 7.46 (dd, J = 17.5, 8.9 Hz, 4H), 7.36 (d, J = 8.4 Hz, 2H), 7.19 (d, J = 8.8 Hz, 2H), 6.61 (d, J = 7.6 Hz, 4H), 3.88 (s, 6H), 3.82 (d, J = 2.2 Hz, 12H). ^{13}C NMR (101 MHz, $CDCl_3$) δ 160.2, 157.6, 155.0, 133. 6, 132.9, 131.5, 129.5, 129.3, 128.6, 127.8, 124.9, 123.7, 119.6, 114.3, 104.7, 99.0, 57.0, 55.6, 55.5. HRMS (APCI) m/z: $[M+H]^+$

calcd for $C_{38}H_{35}O_6$, 587.2434; found, 587.2428. Anal. Calcd for $C_{38}H_{34}O_6$: C, 77.80; H, 5.84. Found: C, 77.82; H, 5.81.

Compound S-2: The same synthetic procedure as **R-2**, yield as yellow solid **(**4.10 g, yield 70%). ^{1}H NMR (400 MHz, Chloroform-*d*) δ 8.09 – 7.97 (m, 4H), 7.46 (dd, *J* = 17.1, 8.9 Hz, 4H), 7.36 (d, *J* = 8.2 Hz, 2H), 7.19 (d, *J* = 8.8 Hz, 2H), 6.61 (d, *J* = 7.5 Hz, 4H), 3.88 (s, 6H), 3.86 – 3.73 (m, 12H). ^{13}C NMR (101 MHz, $CDCl_3$) δ 160.2, 157.6, 155.0, 133.6, 132.9, 131.5, 129.5, 129.3, 128.6, 127.8, 124.9, 123.7, 119.6, 114.3, 104.7, 99.0, 57.0, 55.56, 55.5. HRMS (APCI) m/z: $[M+H]^+$ calcd for $C_{38}H_{35}O_6$, 587.2434; found, 587.2431. Anal. Calcd for $C_{38}H_{34}O_6$: C, 77.80; H, 5.84. Found: C, 77.81; H, 5.83.

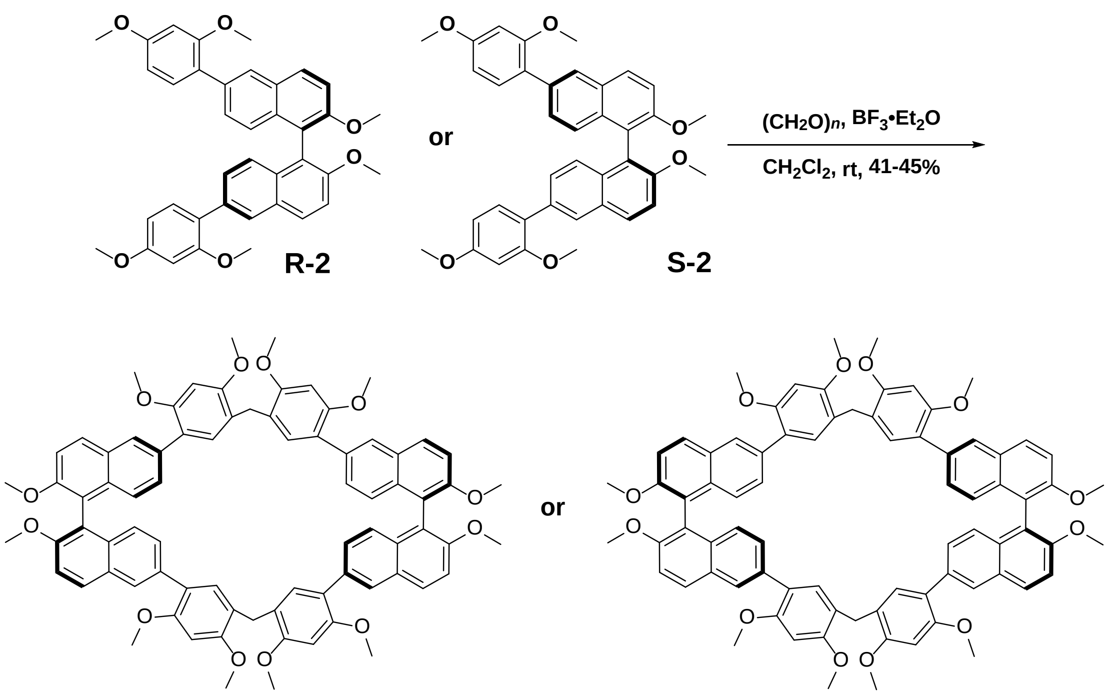

Compound RR-1: To a mixture of **R-2** (1.17 g, 2.0 mmol) and paraformaldehyde (180 mg, 6.0 mmol) in dichloromethane (150 mL) was added boron trifluoride diethyl etherate (0.3 mL, 2.4 mmol). The mixture was stirred at room temperature for 0.5 h. Then the reaction was quenched by the addition of 150 mL water. The organic layer was separated and dried with anhydrous $MgSO_4$. The solvent was removed in vacuo and the residue was separated by column chromatography on silica gel (eluent: 2:1 DCM/Petroleum ether) to give **RR-1** (538 mg, 45%) as a yellow solid. ^{1}H NMR (400

MHz, Chloroform-*d*) δ 7.90 (d, *J* = 9.0 Hz, 4H), 7.84 (s, 4H), 7.39 (d, *J* = 9.0 Hz, 4H), 7.26 (s, 4H), 7.01 – 6.93 (m, 8H), 6.60 (s, 4H), 3.97 (s, 4H), 3.93 (s, 12H), 3.81 (s, 12H), 3.76 (s, 12H). ^{13}C NMR (101 MHz, $CDCl_3$) δ 157.6, 155.8, 154.8, 133.7, 132.7, 132.1, 129.3, 129.2, 128.7, 127.5, 124.6, 122.7, 121.4, 119.6, 114.1, 96.0, 57.0, 56.0, 55.9, 27.8. HRMS (APCI) m/z: $[M+Na]^+$ calcd for $C_{78}H_{68}O_{12}Na$, 1219.4608; found, 1219.4559. Anal. Calcd for $C_{78}H_{68}O_{12}$: C, 78.24; H, 5.72. Found: C, 78.22; H, 5.71.

Compound SS-1. The same synthetic procedure as **RR-2**, yield as yellow solid **(**491 mg, yield 41%). ^{1}H NMR (400 MHz, Chloroform-*d*) δ 7.90 (d, *J* = 9.0 Hz, 4H), 7.83 (s, 4H), 7.39 (d, *J* = 9.0 Hz, 4H), 7.26 (s, 4H), 7.01 – 6.93 (m, 8H), 6.60 (s, 4H), 3.97 (s, 4H), 3.93 (s, 12H), 3.81 (s, 12H), 3.76 (s, 12H). ^{13}C NMR (101 MHz, $CDCl_3$) δ 157.6, 155.8, 154.8, 133.7, 132.7, 132.1, 129.3, 129.2, 128.73, 127.5, 124.6, 122.7, 121.4, 119.6, 114.1, 96.0, 57.0, 55.9, 55.9, 27.8. HRMS (APCI) m/z: $[M+Na]^+$ calcd for $C_{78}H_{68}O_{12}Na$, 1219.4608; found, 1219.4556. Anal. Calcd for $C_{78}H_{68}O_{12}$: C, 78.24; H, 5.72. Found: C, 78.23; H, 5.72.

3. ^{1}H NMR and ^{13}C NMR Spectral of New compounds.

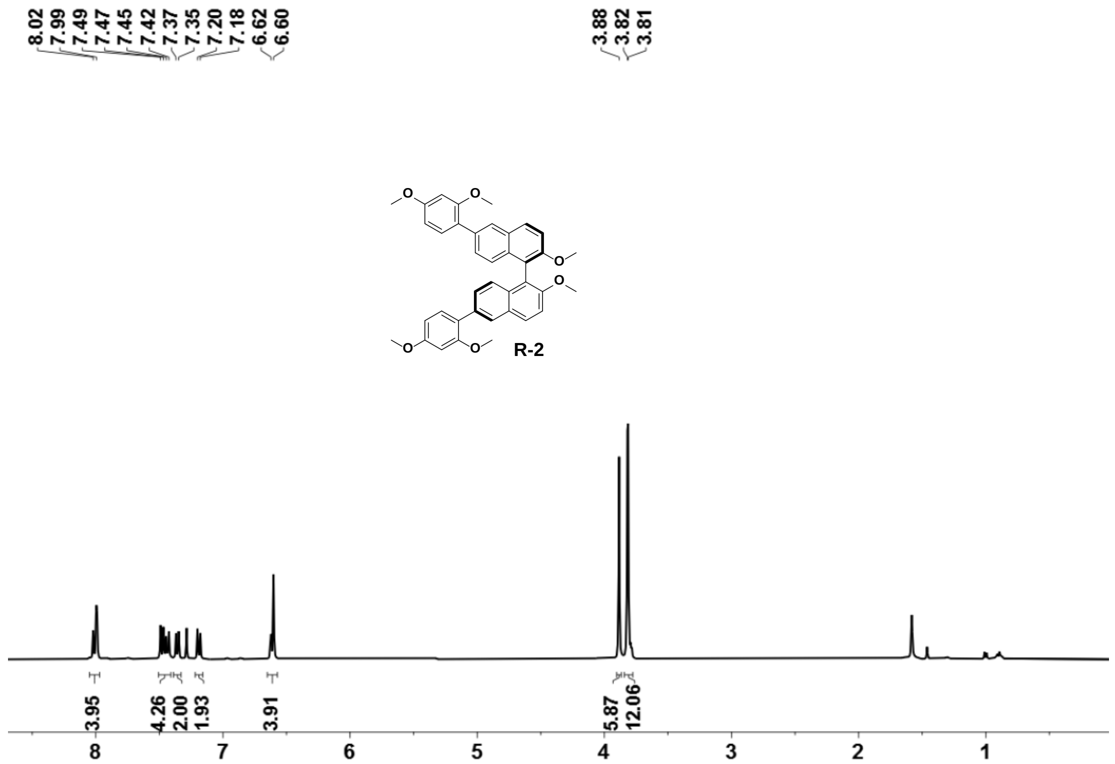

Figure S1. ^{1}H NMR spectrum (400 MHz, $CDCl_3$, 298K) of **R-2**

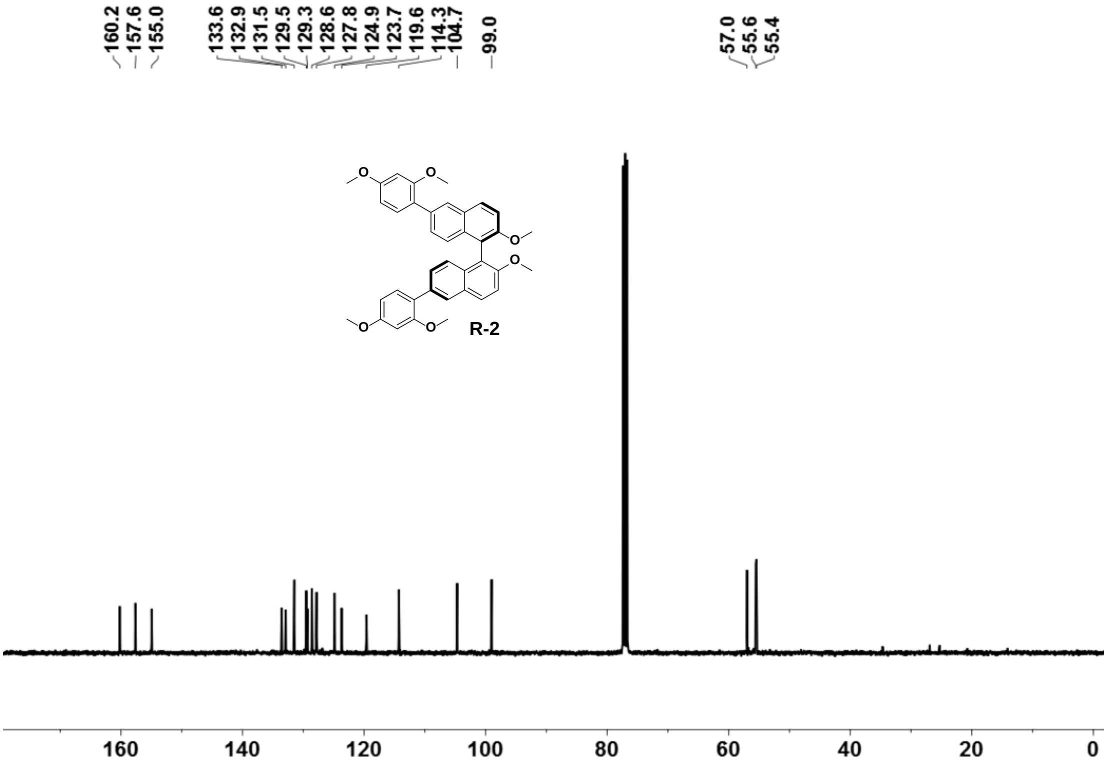

Figure S2.^{13}C NMR spectrum (101 MHz, $CDCl_3$, 298K) of **R-2**

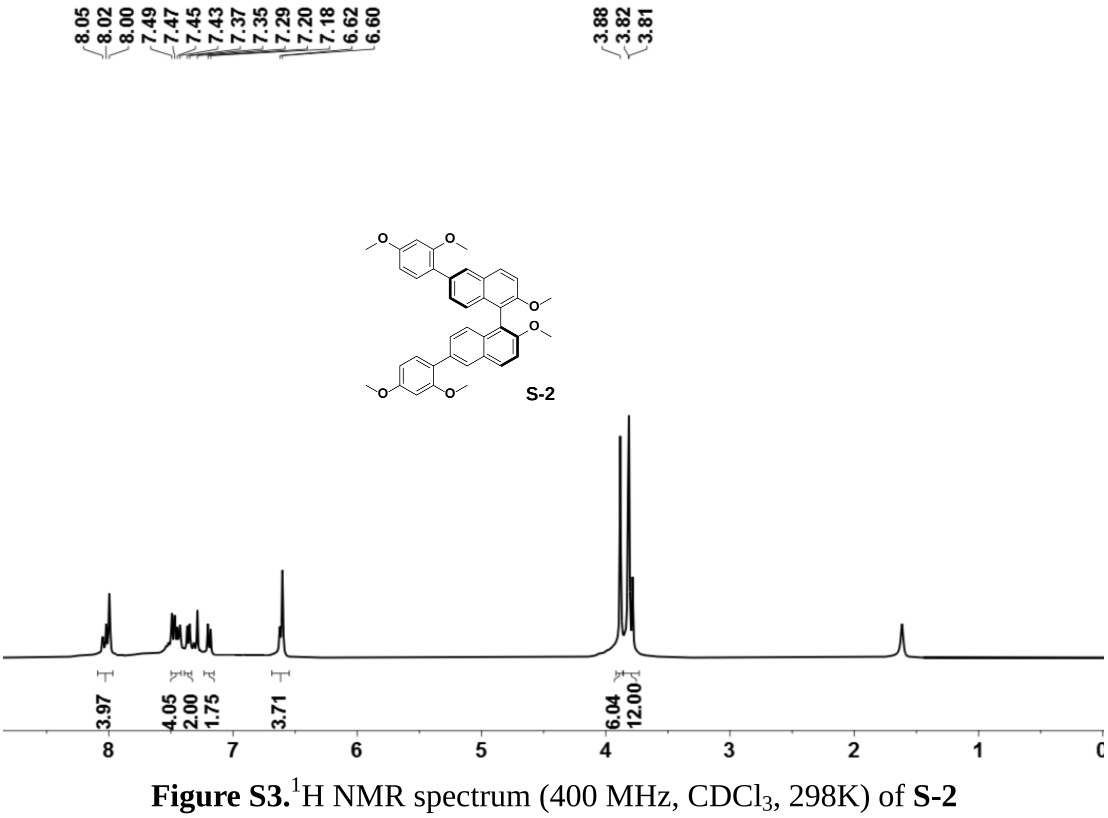

Figure S3.^{1}H NMR spectrum (400 MHz, $CDCl_3$, 298K) of **S-2**

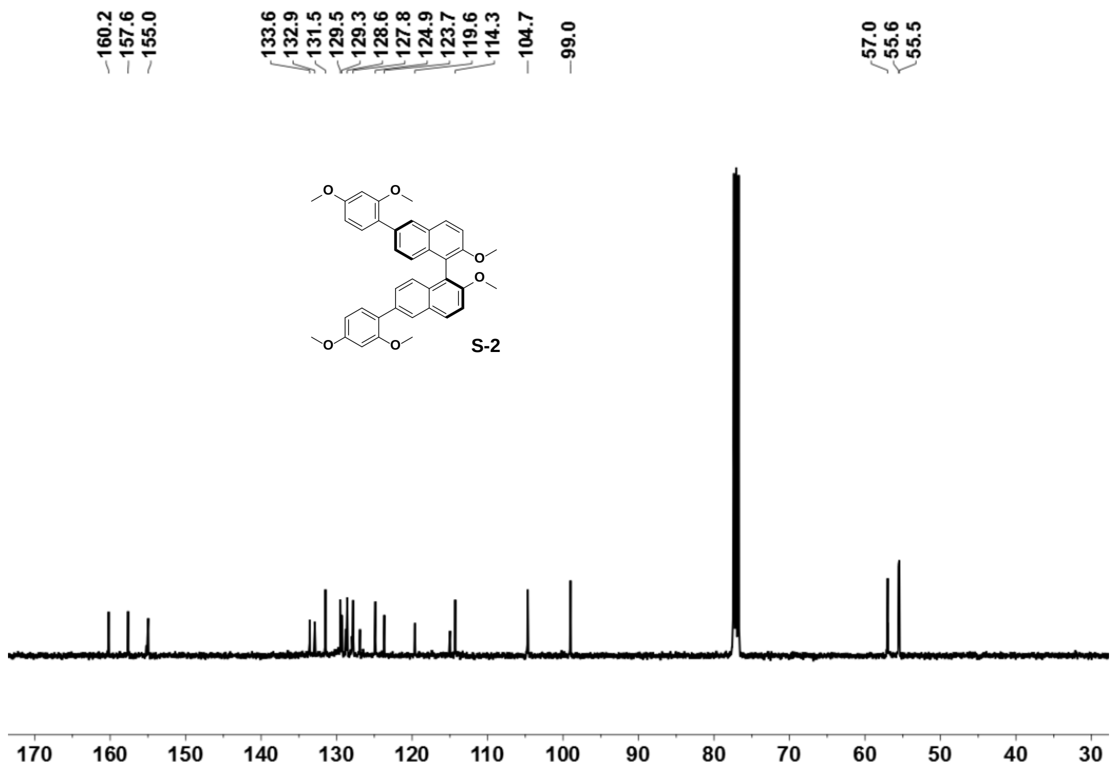

Figure S4.^{13}C NMR spectrum (101 MHz, $CDCl_3$, 298K) of **S-2**

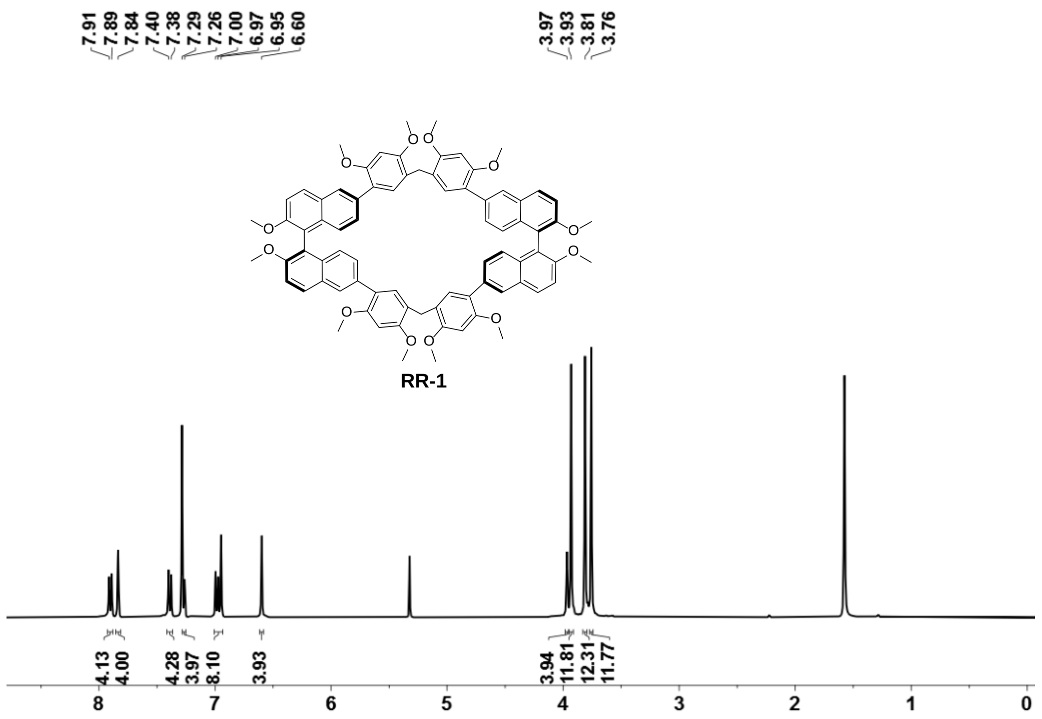

Figure S5.^{1}H NMR spectrum (400 MHz, $CDCl_3$, 298K) of **RR-1**

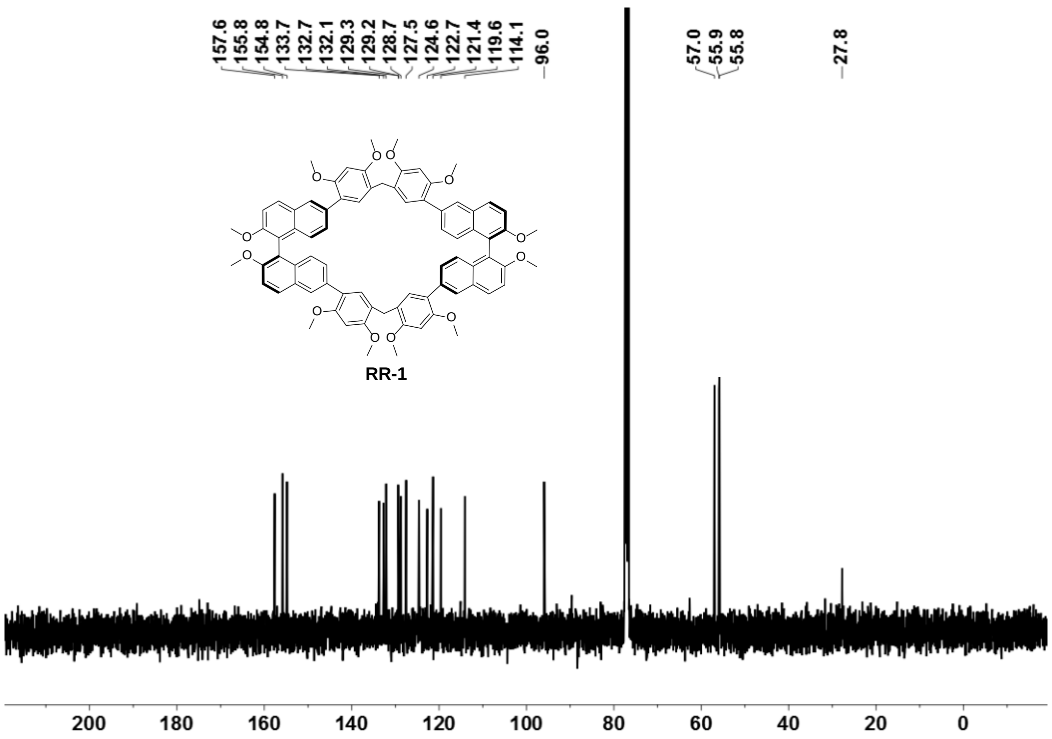

Figure S6. ^{13}C NMR spectrum (101 MHz, $CDCl_3$, 298K) of **RR-1**

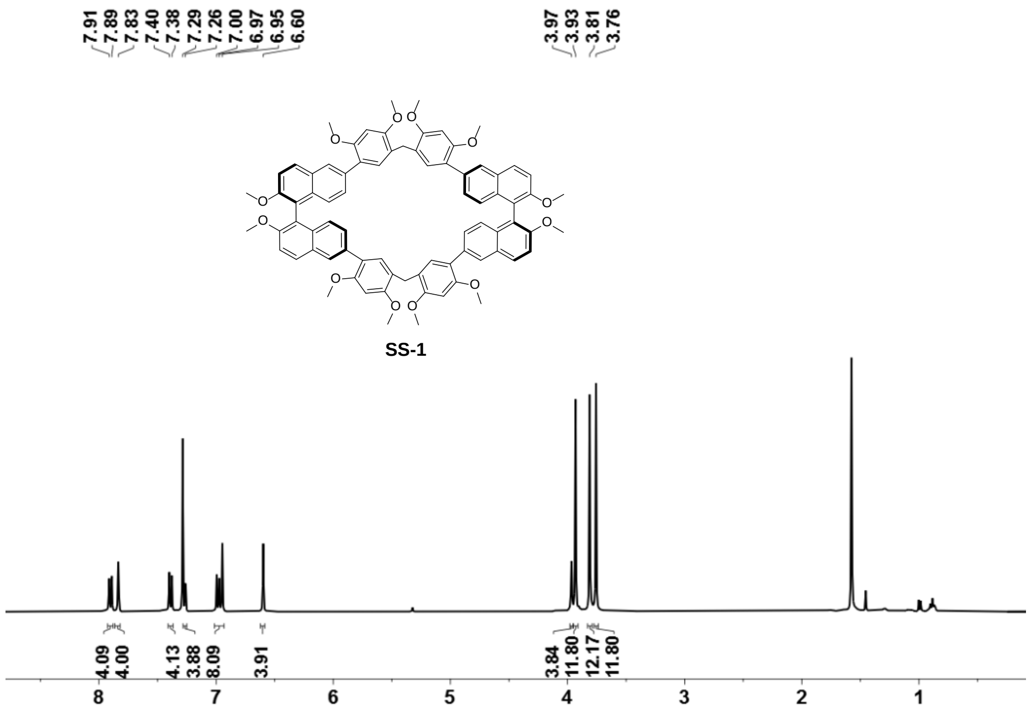

Figure S7. ^{1}H NMR spectrum (400 MHz, $CDCl_3$, 298K) of **SS-1**

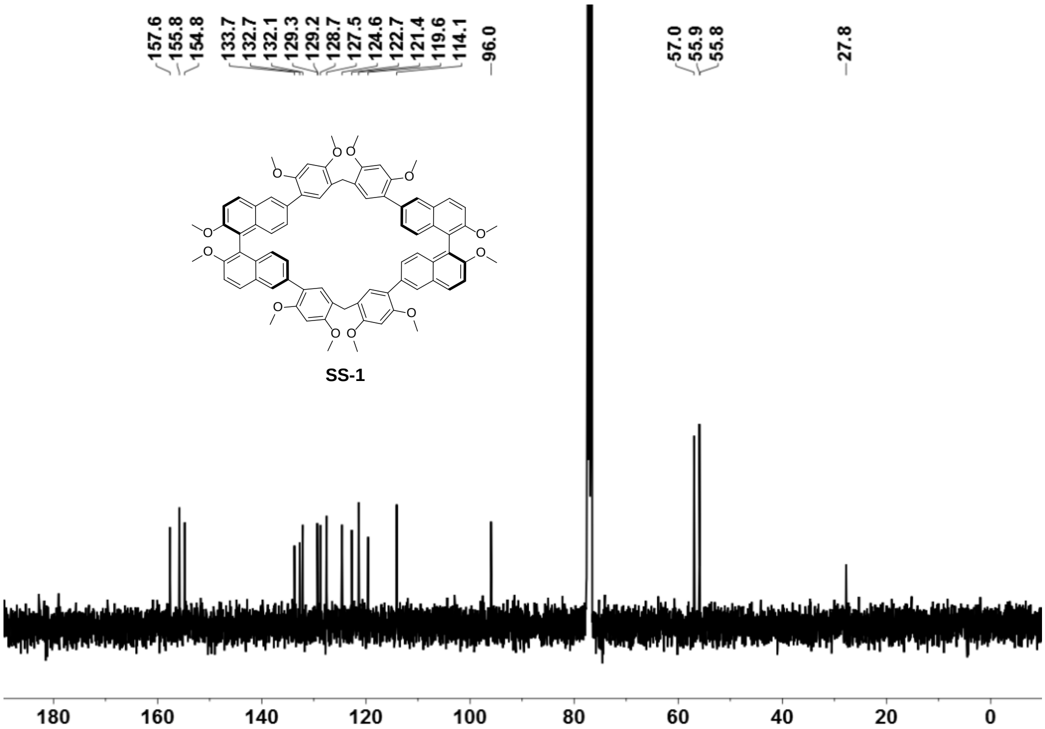

Figure S8. ^{13}C NMR spectrum (101 MHz, $CDCl_3$, 298K) of **SS-1**

4. ^{1}H NMR studies of Complexation of the Host and Guest

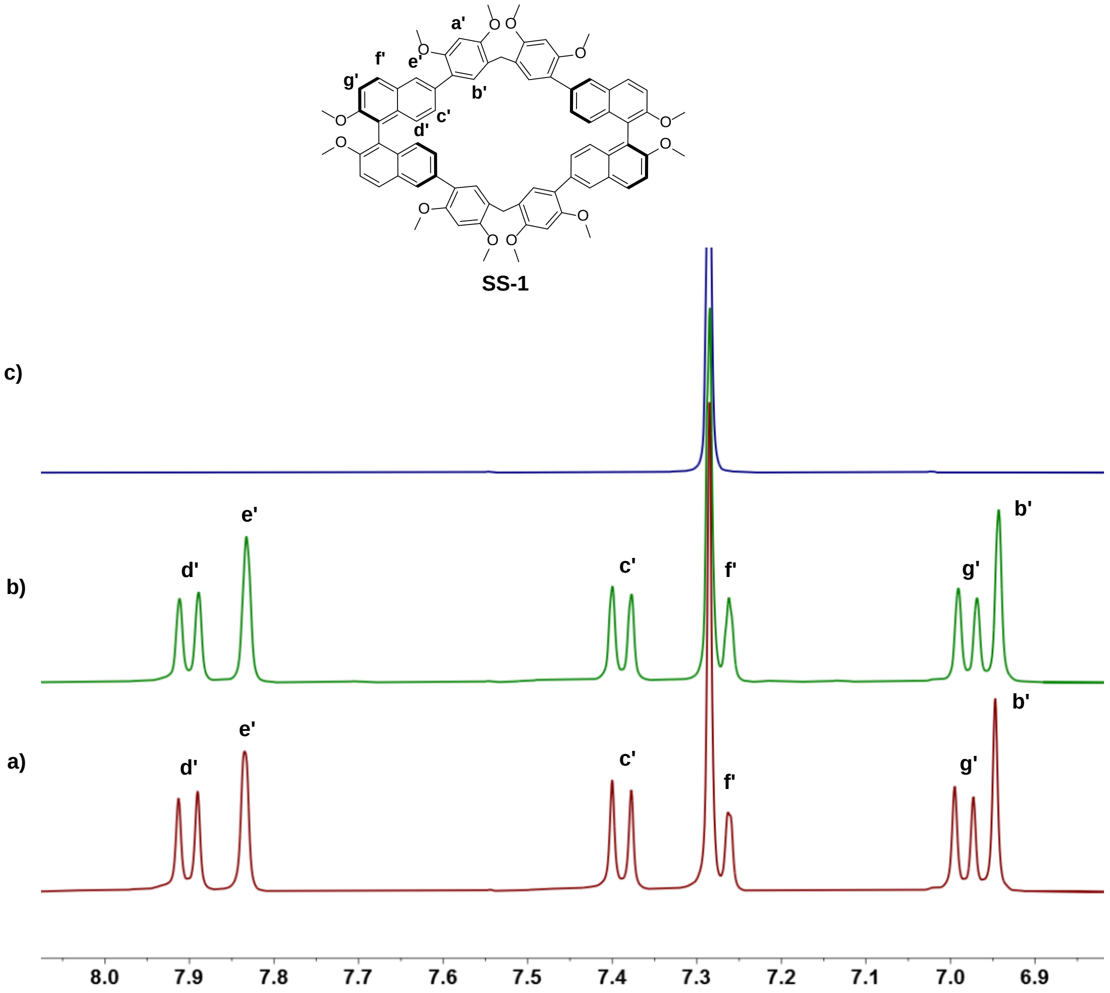

Figure S9. Partial ^{1}H NMR spectra (400 MHz, $CDCl_3$, 298 K) of (a) free **SS-1**, (b) **SS-1** and 1.0 equiv. of TBAI, and (c) free TBAI. [**SS-1**]$_0$ = 4.0 mM.

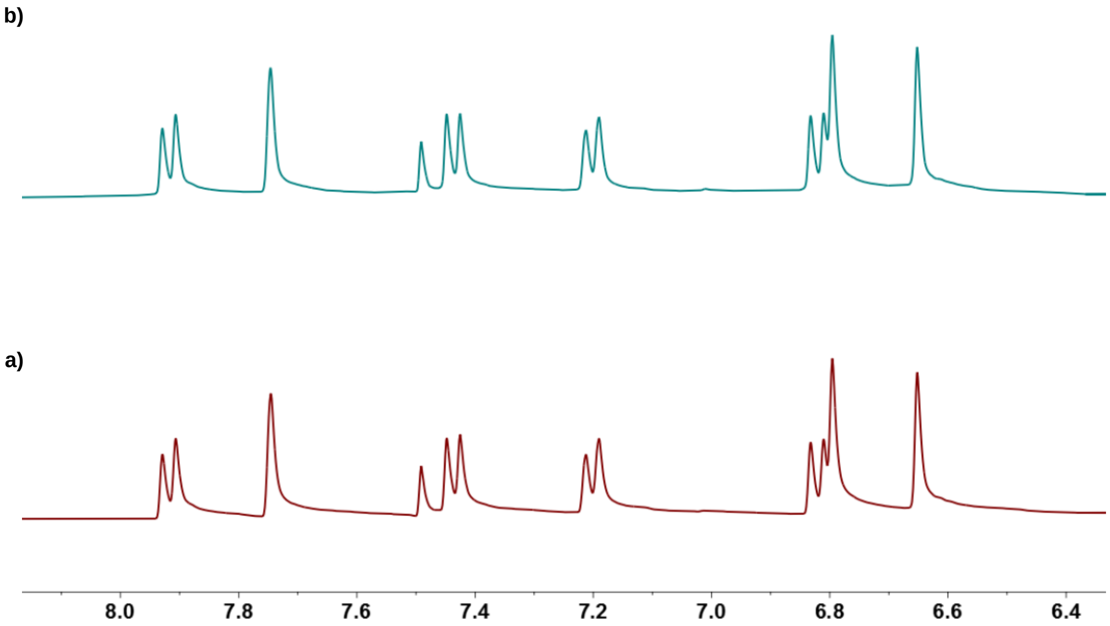

Figure S10 Partial ^{1}H NMR spectra (400 MHz, $CDCl_3$, 298 K) of (a) free **RR-1**, (b) **RR-1** and 1.0 equiv. of tetramethylammonium hexafluorophosphate. [**RR-1**]$_0$ = 4.0 mM.

5. Determination of the Association Constants of the Complexes

In the ^{1}H NMR titrations, $CDCl_3$ was chosen to dissolve the host and the guests. Chemical shifts were reported in parts per million (*ppm*). By a mole ratio plot, each stoichiometry was determined. Titration curve-fitting and association constant values were calculated by employing the BindFit program developed by Prof. Pall Thordarson of UNSW. 1:1 Binding stoichiometry was chosen in the BindFit program. This program employs a nonlinear least-squares regression analysis and is available free of cost online through the following link: http://supramolecular.org.

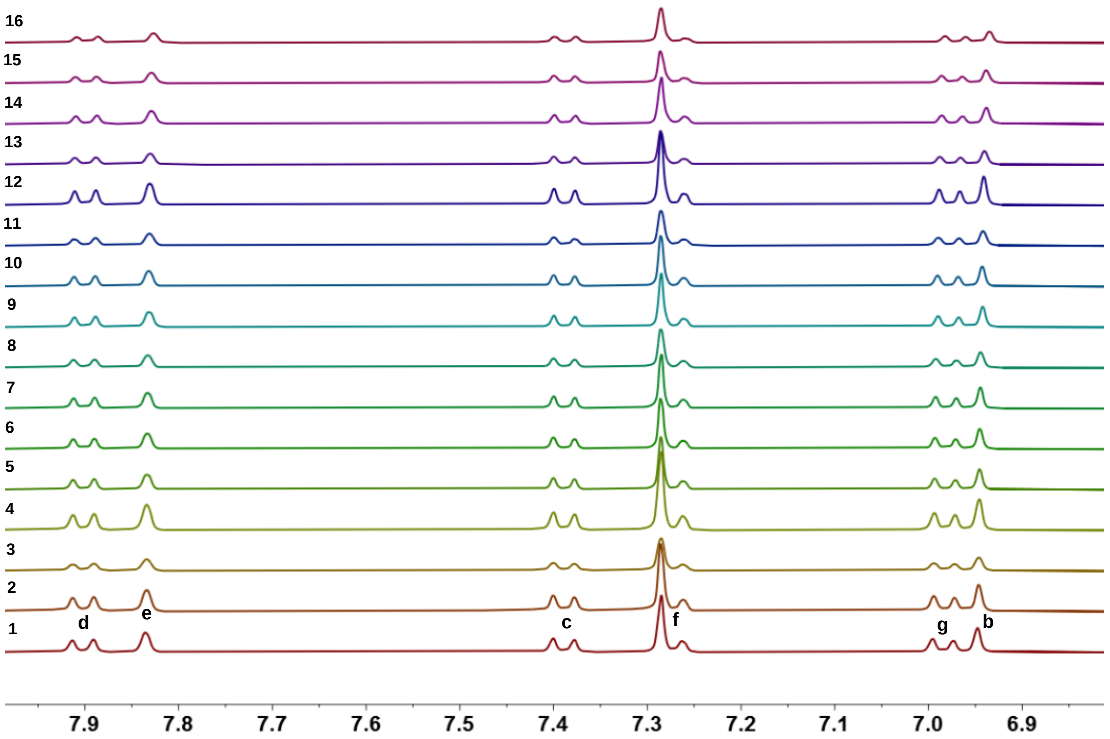

Figure S11. ^{1}H NMR spectra (400 MHz, $CDCl_3$, v/v, 298 K) of **RR-1** at a concentration of 4.0 mM with different concentrations of TBAI: (1) 0.00 mM; (2) 0.8 mM; (3) 1.2 mM; (4) 1.6 mM; (5) 2.0 mM; (6) 2.4 mM; (7) 2.8 mM; (8) 3.2 mM; (9) 3.6 mM; (10) 4.0 mM; (11) 4.8 mM; (12) 5.6 mM; (13) 6.4 mM; (14) 8.8 mM; (15) 12.0 mM; (16) 40.0 mM.

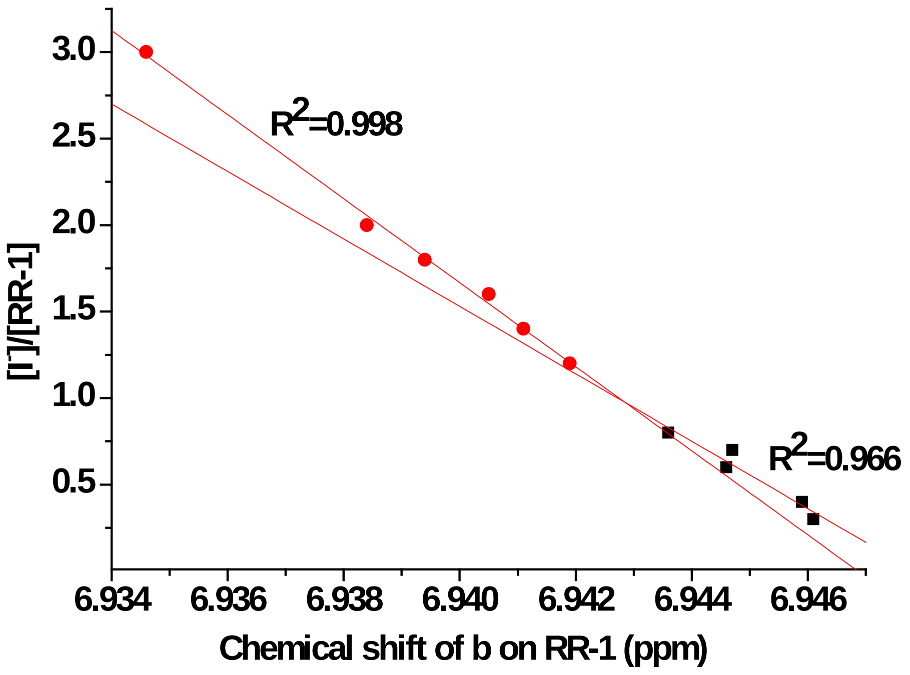

Figure S12. Mole ratio plot of the complexation of **RR-1** and TBAI in $CDCl_3$ at 298 K.

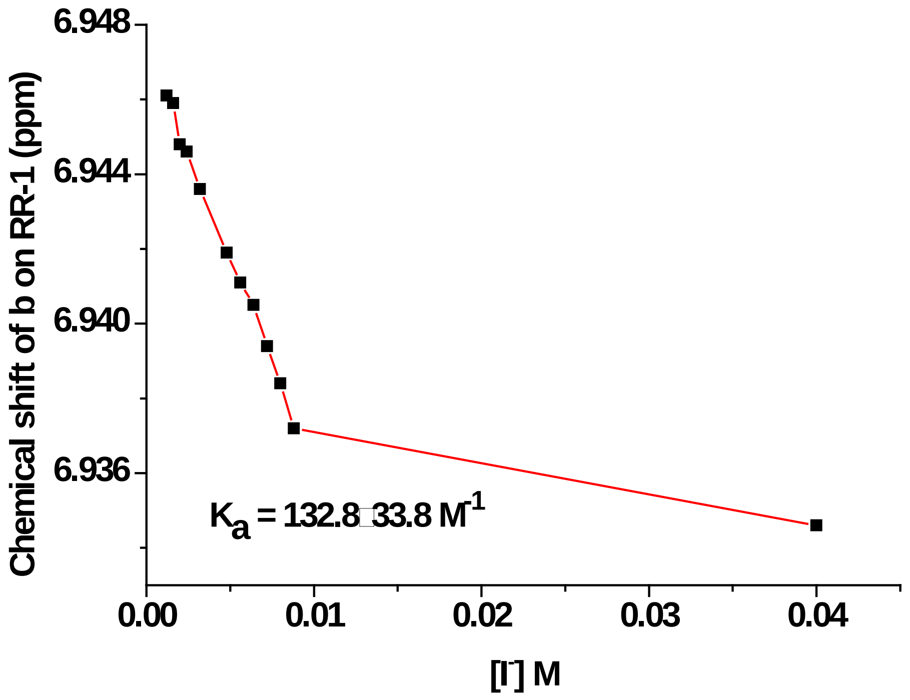

Figure S13. Plot of chemical shift (ppm) for the b of **RR-1** and TBAI in $CDCl_3$ at 298 K.

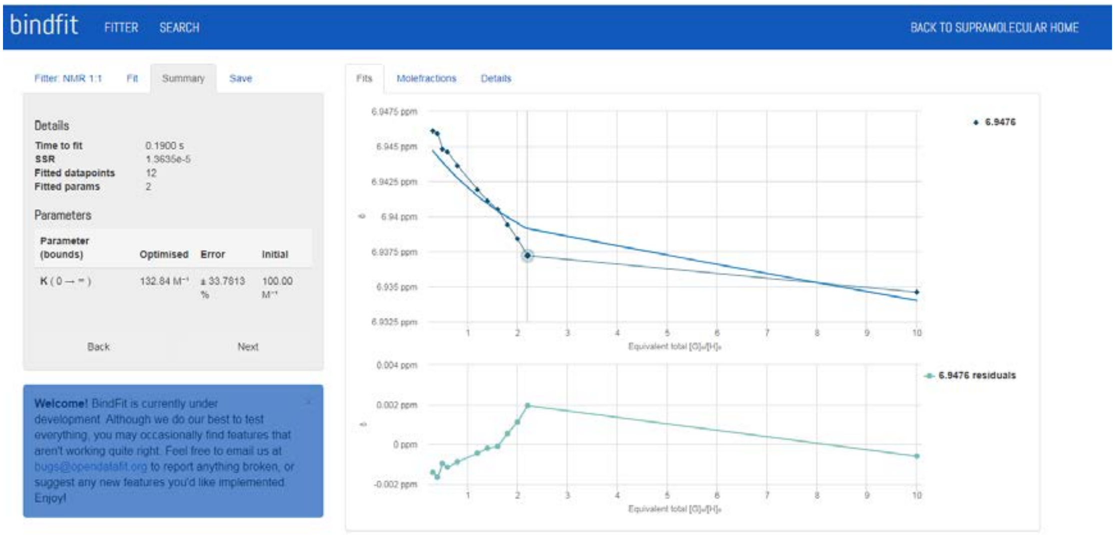

Figure S14. Nonlinear least-square analysis of the ^{1}H NMR binding data corresponding to the formation of [**RR-1**•I]$^{-}$ complex. The data were fitted to a 1:1 binding model to give K_a = 132.8 ± 33.8 M^{-1}. The residual distribution is shown below the binding isotherm. All solid lines were obtained from non-linear curve-fitting to a 1:1 binding model using the www.supramolecular.org web

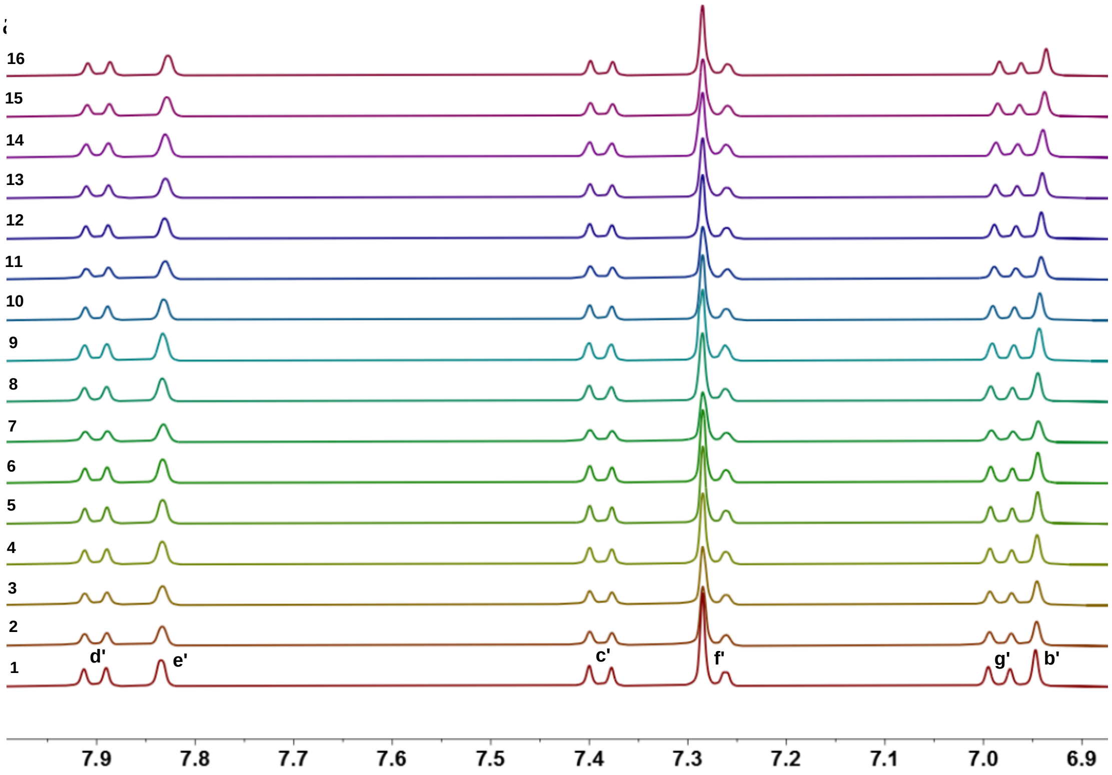

Figure S15. ^{1}H NMR spectra (400 MHz, $CDCl_3$, v/v, 298 K) of **SS-1** at a concentration of 4.0 mM with different concentrations of TBAI: (1) 0.00 mM; (2) 0.8 mM; (3) 1.2 mM; (4) 1.6 mM; (5) 2.0 mM; (6) 2.4 mM; (7) 2.8 mM; (8) 3.2 mM; (9)

3.6 mM; (10) 4.0 mM; (11) 4.8 mM; (12) 5.6 mM; (13) 6.4 mM; (14) 8.8 mM; (15) 12.0 mM; (16) 40.0 mM.

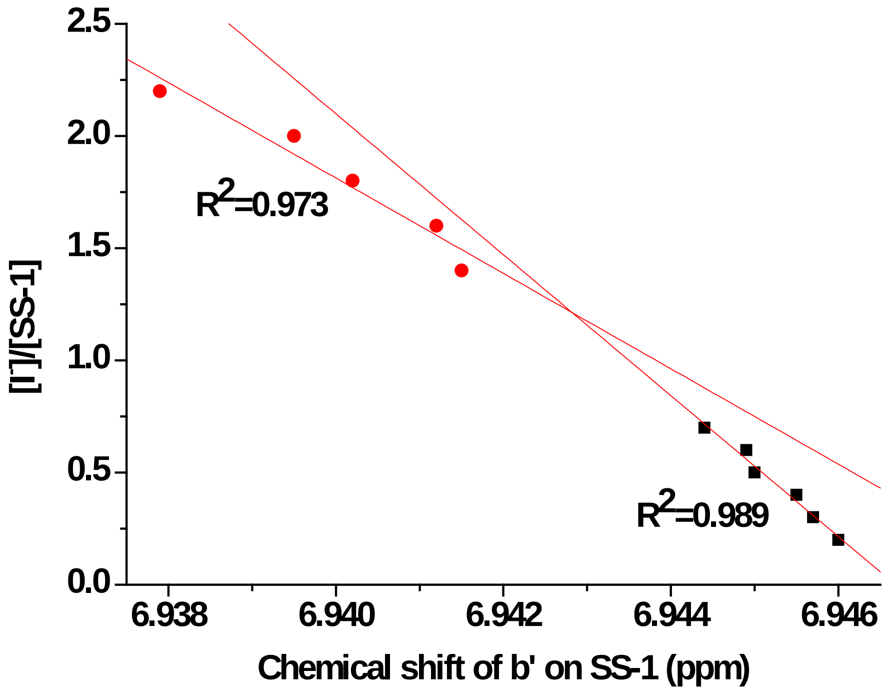

Figure S16. Mole ratio plot of the complexation of **SS-1** and TBAI in $CDCl_3$ at 298 K.

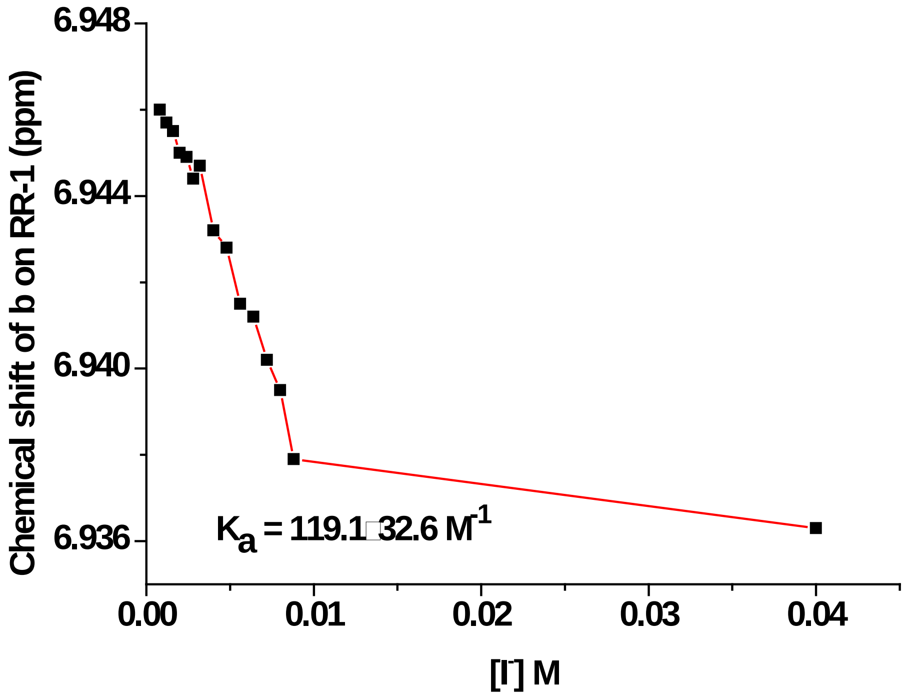

Figure S17. Plot of chemical shift (ppm) for the b'of **SS-1** and TBAI in CDCl3 at 298 K.

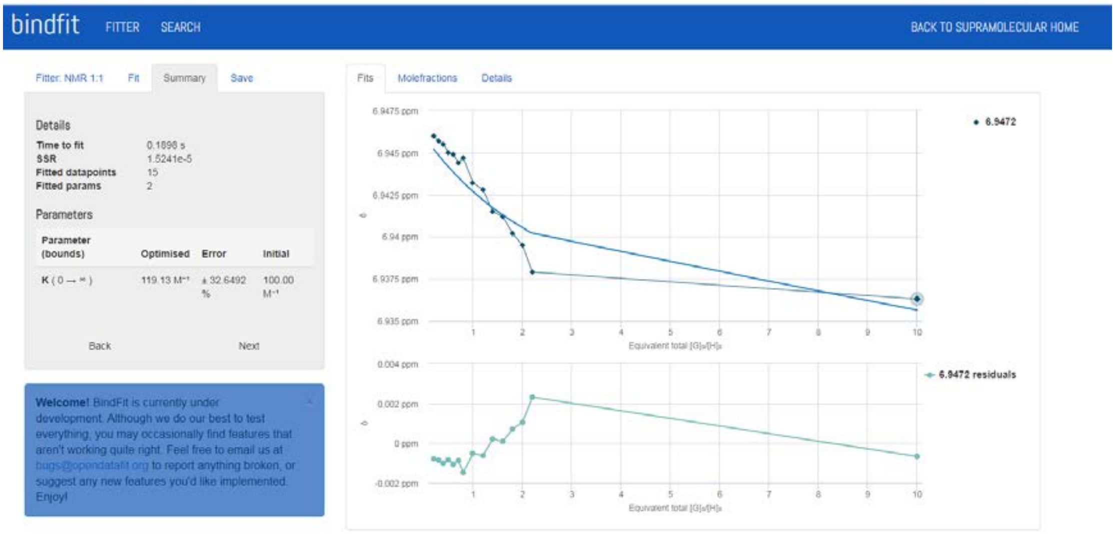

Figure S18. Nonlinear least-square analysis of the ^{1}H NMR binding data corresponding to the formation of [**SS-1•**I]$^{-}$ complex. The data were fitted to a 1:1 binding model to give K_a = 119.1 ± 32.6 M^{-1}. The residual distribution is shown below the binding isotherm. All solid lines were obtained from non-linear curve-fitting to a 1:1 binding model using the www.supramolecular.org web applet.

6. ESI MS studies of new compouds and complexes

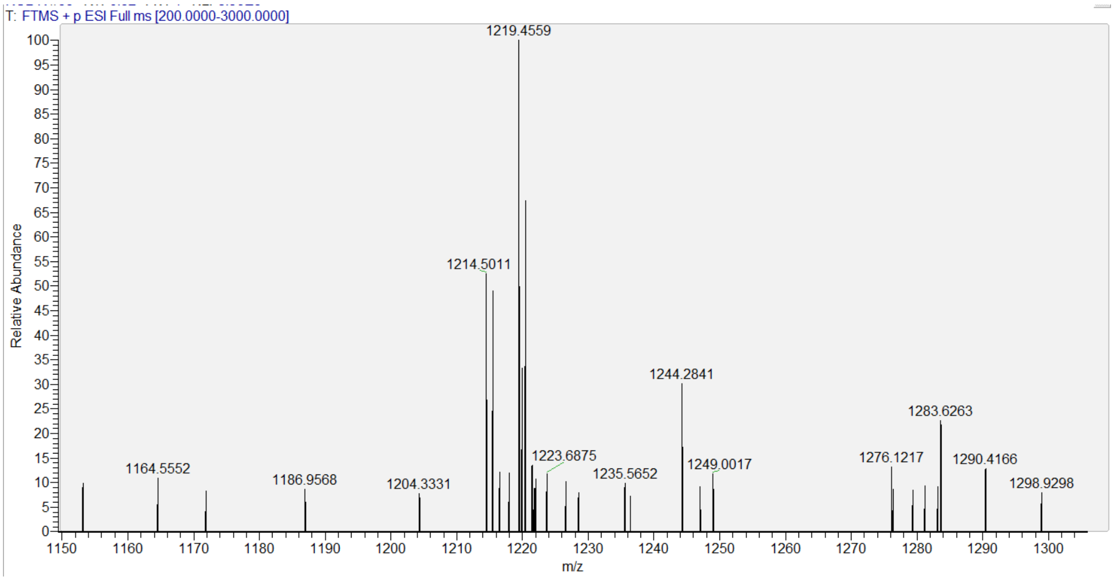

Figure S19. ESI Spectrum of **RR-1**

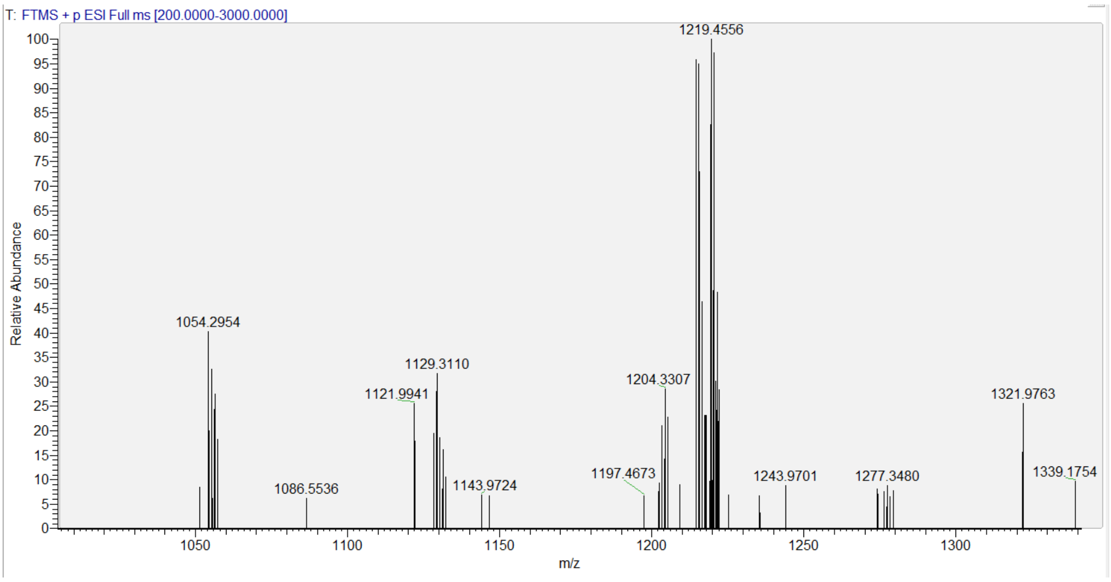

Figure S20. ESI Spectrum of **SS-1**

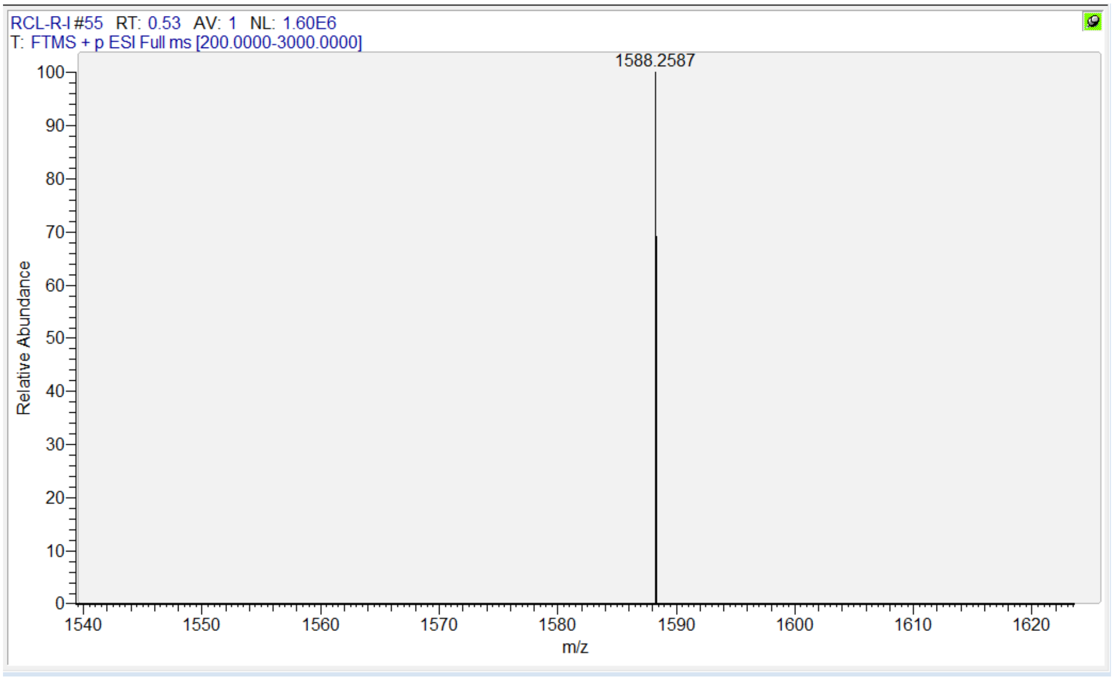

Figure S21. ESI Spectrum of **RR-1**/iodide

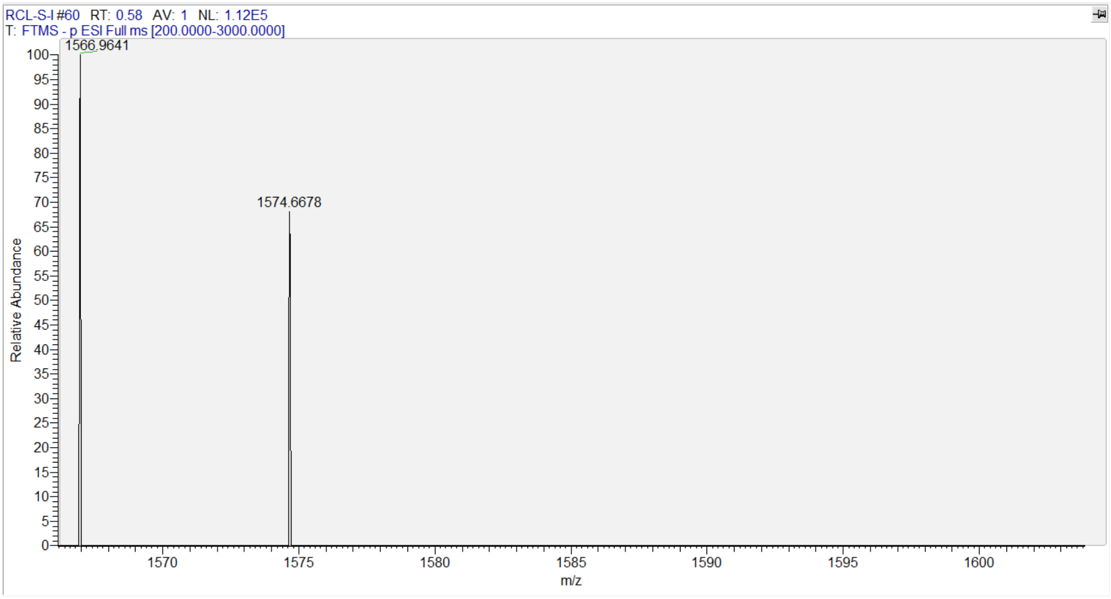

Figure S22. ESI Spectrum of **SS-1**/iodide

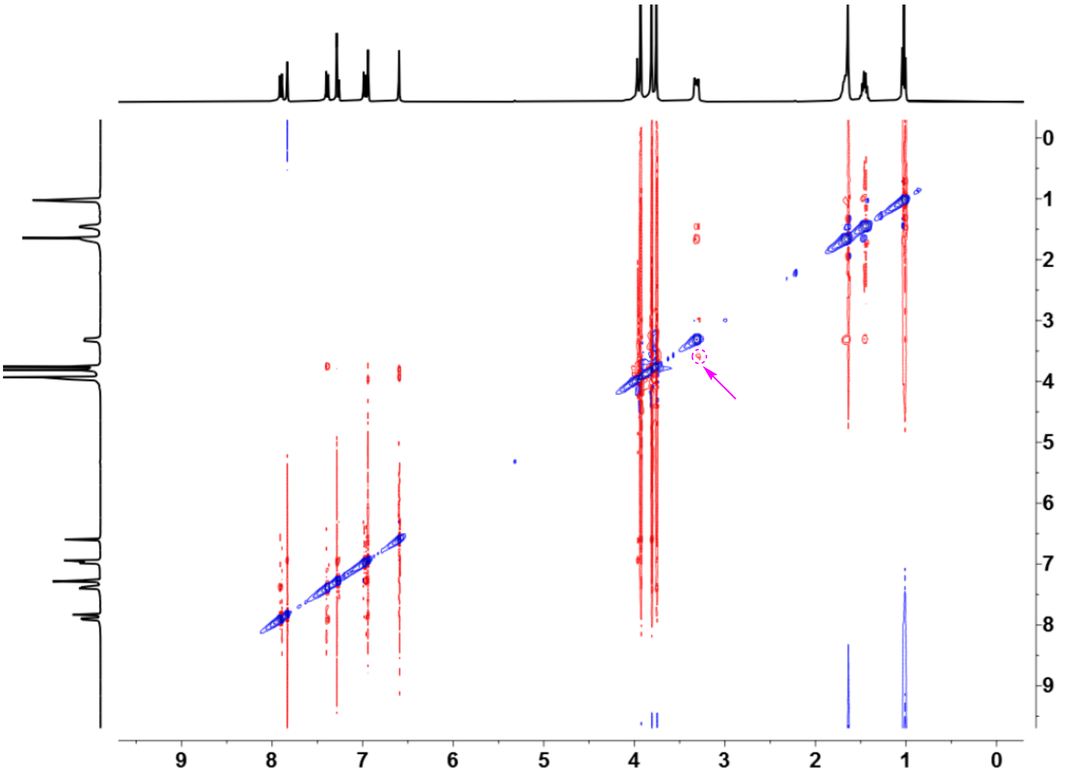

Figure S23. ^{1}H-^{1}H ROESY spectrum (400 MHz, $CDCl_3$, 298 K) of **RR-1**/iodide

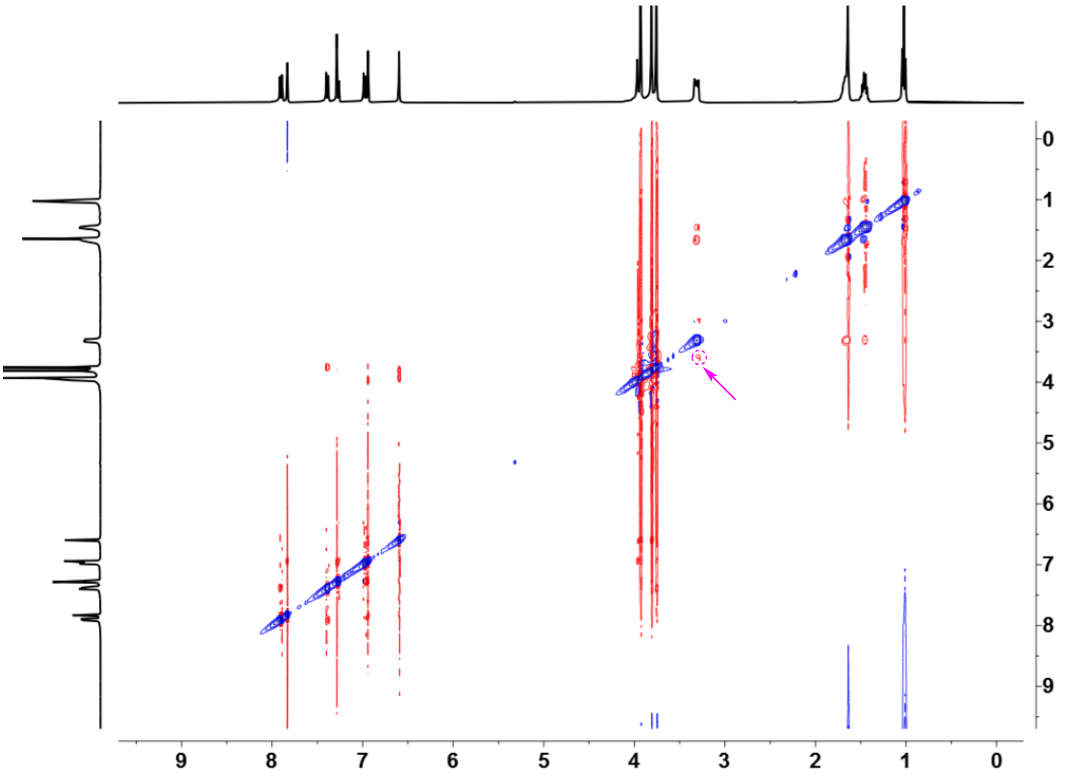

Figure S24. ^{1}H-^{1}H ROESY spectrum (400 MHz, $CDCl_3$, 298 K) of **SS-1**/iodide
